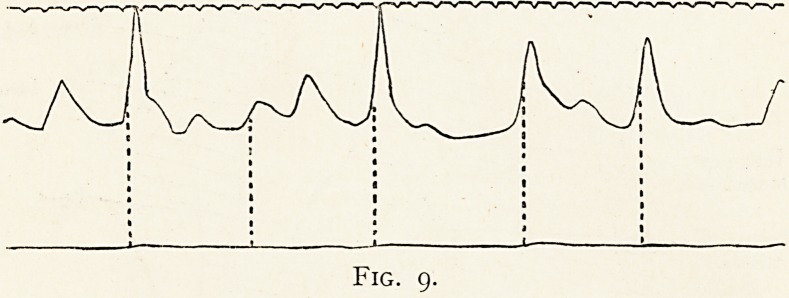# The Simulation of Aortic Aneurysm by Some Other Aortic and Cardiac Diseases
1Read at a meeting of the Bristol Medico-Chirurgical Society on June 14th.


**Published:** 1914-03

**Authors:** Carey Coombs

**Affiliations:** Assistant Physician to the Bristol General Hospital


REFERENCES.

### THE SIMULATION OF AORTIC ANEURYSM BY SOME OTHER AORTIC AND CARDIAC DISEASES.[1]

BY

CAREY COOMBS, M.D., M.R.C.P. Lond.,

*Assistant Physician to the Bristol General Hospital.*

THERE are two sides to the diagnosis of serious disease; its discovery on the one hand, and its exclusion on the other. Aortic aneurysm, for example, is of such gravity that it ought never to be overlooked; yet it is of some importance that a diagnosis of aneurysm should never be made except when it is actually present, for this diagnosis is tantamount to the dismissal of the patient from active life. There are certain diseases of intrathoracic organs of a much less

[1] Read at a meeting of the Bristol Medico-Chirurgical Society on June 14th.

desperate nature than aneurysm, which may imitate to a certain point the clinical picture of that disease. It is with cardiac and aortic lesions of this type that my paper is specially concerned.

The introduction of skiagraphy has not only afforded a new means for the detection of aneurysm, but also given much new knowledge of *general aortic dilatation*. Many cases of this type were regarded as aneurysm in the pre-skiagraphic days, and no wonder. Two factors are mainly concerned in bringing about diffuse enlargement of the aorta, disease weakening its walls and increased pressure within its lumen. The combination of these two factors in varying proportion is seen in three clinical classes. In the first the patient is suffering from syphilitic aortitis, in itself a pre-aneurysmal state, with or without disease of thə aortic valves. The first diagram (Fig. 1) illustrates the physical signs in a woman aged 66, with definite evidence of tertiary syphilis. During the ten years she has been under observation her physical signs have become less obvious, and the X-ray screen fails to discover any sac. The next plate (Fig. 2) shows the enormously dilated aorta, the seat of diffuse syphilitic inflammation, from a man aged 44, who had syphilis at 31. He showed marked signs of aortic regurgitation, with vigorous pulsation in the episternal notch. Absence of pressure signs helped us to exclude aneurysm. The second group of cases

FIG. 1.

( ⁝ = Area of aortic dulness (leftward border ill defined).
● = Maximal intensity of systolic bruit.
× = Area of localised pulsation.

comprises patients with high arterial tension and arterio-sclerosis; these rarely reach such a pitch as to imitate aneurysm, but I include one diagram (Fig. 3) of a woman of 48 recently seen, in whom a loud, deep-toned second sound, a palpable diastolic shock, and an enlargement of some of the superficial cervical veins were associated with a very high

FIG. 3.

= Position of enlarged veins in neck.

= Area of diastolci shock.

+ = Site of maximum accentuation of second sound.

F. 48 with high arterial tension and secondary aortic dilatation.

FIG. 4.

= Outline of cardio-aortic dulness.

O = Maximum aortic pulsation.

□ = Maximum diastolic murmur.

× = Maximum ventricular pulsation.

tension but no aneurysm. In the third group may be placed cases of aortic dilatation associated with sclerosis and insufficiency of the aortic valves following rheumatic carditis; these cases have been wrongly described as "rheumatic aneurysms." The accompanying diagram (Fig. 4) portrays the physical signs remarked in a man of 37, who had aortic regurgitation following rheumatic infection. I have watched him for seven years, and Dr. F. G. Bergin has taken several X-ray pictures of him. These show a diffusely enlarged aorta but no sac. In the syphilitic group, disease of the aortic wall is mainly responsible for the dilatation ; in the atheromatous both factors are combined ; while in the third group, though rheumatism does some slight injury to the aortic wall, the principal factor is over-distension by the large mass of blood thrown into the aorta at each ventricular systole.

In all these types of aortic dilatation some of the physical signs of aneurysm are produced, as in the cases quoted. On either side of the manubrium there is a strip of dulness extending as far as two fingers' breadth beyond the sternal margin on the right side and rather less on the left. Pulsation is visible at the inner ends of the second and third right intercostal spaces, and is sometimes so distinctly localised as to look expansile, though it is not actually so. The aortic second sound is often so much accentuated, even when it is followed by a diastolic bruit, as to arouse suspicion of the presence of an aneurysm. In a few cases the intercostal veins are a little enlarged in the upper spaces on the left side, and this is apt to lend colour to the diagnosis of aneurysm. And finally, if the aortic valves are incompetent, hæmoptysis may occur.

Pressure signs are also imitated in some instances by *aortitis of localised distribution* implicating branch arteries or neighbouring structures. This point may best be illustrated by the following cases. A man of 48 came on to me from the surgical out-patient department of the Hospital complaining of pain in the chest following strain. I have had many opportunities of seeing him, both as an out-patient and afterwards in the wards. His chief symptoms were pain in the chest on stooping, and curious attacks of loss of power on exertion (*e.g.* during shaving) in the right arm. This loss of power was brief; it was accompanied by cramp-like contractions of the muscles resulting in involuntary pronation of the forearm and flexion of the fingers, and it was painful. The physical signs were those of aortic regurgitation; over the upper chest a diffuse systolic heave was discernible, and the possibility of an aneurysm being present was further suggested by marked asymmetry of the temporal and carotid pulses as illustrated in the accompanying tracing (Fig. 5), which shows asynchronism in the onset of the two waves. This difference was not discernible in the radial pulses. Skiagraphy demonstrated quite clearly, however, the absence

FIG. 5.

of any sign of aneurysmal bulge, and the subsequent course of the case has confirmed this. The explanation of the phenomenon probably lies in implication of the orifices of the arterial trunks springing from the aortic arch in the syphilitic or atheromatous process, leading to their partial obstruction. This was actually found in one of the cases of syphilitic aortitis with unequal pulses, reported by Laignel-Lavastine and Vinlich,[1] and I have several times seen asymmetry of the radial pulses in a similar disease, endarteritis obliterans. Another case of aortitis was that of a man aged 39 who was admitted to Dr. Parker's ward when I was temporarily in charge, on account of dyspnœa and swelling of the feet of a month's duration, with a fortnight's œdema of the back and chest. There was albumin in the urine ; the face and hands were faintly cyanosed, and there was a remarkable pulsatile distension of the veins in the arms and the chest wall. Beyond a diffuse diastolic shock, there were no other distinct signs. Sudden symptoms of embolism of the left brachial artery with those of acute cardiac failure terminated the case. Aneurysm was suspected on account of the venous distension ; but skiagraphy excluded this, and the lesion found *post-mortem* was a band of acute aortitis encircling the intrapericardial aorta, and leading to formation of vegetations which projected into its lumen (Fig. 6). Mr. Scott-Williamson, who performed the autopsy, suggested that the œdema and venous distension were due to pressure upon the superior vena cava by the aorta, which must have been distended on the cardiac side of the inflammatory stricture, added to which some weight must be allowed to the extraordinary widening of the tricuspid orifice which was found *post-mortem.*

In cases such as these, and especially where the veins are enlarged and the radial pulses asymmetrical in time or volume, it is often impossible to exclude the presence of aneurysm except by means of skiagraphy. Dr. Bergin and I have examined a number of patients presenting such physical signs, and we find that it is essential to screen the chest from both back and front, and also to look at the aorta in profile, using the right anterior oblique position. In diffuse dilatation the aortic shadow is enlarged in every direction ; in aneurysm, a definite localised bulge is seen.

In the other class of case to which attention must be drawn it is not the aorta but the heart itself that furnishes a lesion capable of simulating aneurysm. In a very large number of cases of chronic heart disease it is the left auricle that fails and suffers from the effect of mechanical disability.

Nowhere is this more noteworthy than in chronic rheumatic disease of the heart ; sclerosis of the mitral cusps is a constant feature of such cases, whether accompanied or not by other permanent injuries, and this practically always implies some degree of obstruction to the emptying of the left auricle. The musculature of this chamber, never very powerful, soon permits of dilatation, to which hypertrophy may or may not be added. The consequence is an *enlargement of the left auricle,* so disproportionate that in some of its pressure effects at any rate it may be regarded as a kind of intrathoracic tumour. The best known of these pressure effects is paralysis of the left vocal cord, due to compression of the left recurrent laryngeal nerve. There are about fifty examples of this on record, to which I have to add one and a probable second.

My first patient was a newly-married woman of 27, referred to me at the Hospital by Mr. Lacy Firth, who had discovered the laryngeal palsy and sought for a cause within the chest. She had had chorea several times, between the ages of 9 and 14 ; her dyspnœa, which was quite severe when I first saw her, had increased rapidly of late. The cardiac signs were those of mitral stenosis coupled with great enlargement of the left ventricle, and hyperæmia of the lungs. Under treatment the dyspnœa became less, but the laryngeal symptoms did not alter. Some time later I saw her at her home with Dr. C. M. Phillips of Brislington. She was about five months pregnant, and had become intensely and progressively dyspnœic. We decided that she could not bear any further increase in her abdominal pressure ; she was accordingly taken into hospital and labour was induced by Dr. Roger Wright, who was house physician at the time, Mr. Rayner supervising. This passed off successfully, but she did not improve much, and died at home a few days later of cardiac failure, autopsy being impossible.

The second patient, aged about 45, had been sent to

Fig. 2.

Fig. 6.

Dr. Watson-Williams suffering from hoarseness. He found this to be due to paralysis of the left recurrent laryngeal nerve. and very kindly invited me to see whether any light could be thrown on its cause. Since her teens she had suffered from dyspnœa ; there was no history of any rheumatic manifestation, but she remembered that at 17 she was forbidden to go up hills because of her heart. She had, however, led a very active life, though more and more restricted by dyspnœa. The chief features of the case when I saw her (she had then but lately returned from Nauheim) were dyspnœa and cyanosis, bubbling râles at both bases, great increase in the transverse diameter of the heart, accentuation of the first sound at the apex, preceded by what might possibly be described as a very short presystolic bruit, and increase in the second sound at the base. Her pulse was small but regular ; what added to the possibilities of error was that on the left side she had a very small radial artery and a very large superficialis volæ branch, the result being a false inequality of the radial pulses. The X-ray photographs which Dr. Bergin took proved the absence of aneurysm.

In the first of these two cases there is no doubt whatever that the pressure of an enlarged left auricle was responsible for the laryngeal palsy. The second case is probably susceptible of a similar explanation. The patient had been known to have had cardiac disease at 17, which appears to have been acquired and not congenital if we may judge from the history, and ninety per cent. of all cases of heart disease acquired before 20 are rheumatic in type. Add to this the fact that the physical signs, though not pointing directly to mitral stenosis, proved an enlargement of the heart both to the right and the left—a state of affairs highly characteristic of post-rheumatic disease—and it is difficult to oppose any strong argument to the diagnosis of rheumatic disease of the heart, left auricular enlargement, and compression of the left recurrent laryngeal nerve. It has of late been suggested that in some of these cases at any rate the recurrent laryngeal nerve becomes implicated in a mediastino-pericardial inflammation, but the weight of *post-mortem* evidence is in favour of the auricular theory. Fetterolf and Norris,[2] indeed, consider from a study of frozen sections that the auricle does not directly compress the nerve, but that it lifts up the left branch of the pulmonary artery in such a way as to nip the nerve between that artery and the aorta. The diagram (Fig. 7) is from one of their photographs of dissections.

FIG. 7.

DIAGRAM OF RELATION BETWEEN LEFT AURICLE AND LEFT RECURRENT LARYNGEAL NERVE (after Fetterolf and Norris).

1. Left recurrent laryngeal nerve.
2. Aorta.
3. Pulmonary artery at bifurcation.
4. Left auricle.
5. Left ventricle.

Other pressure effects of the enlarged left auricle are less familiar. Several writers have described inequalities of the radial pulses which they refer to this cause, and others have ascribed evidences of compression of the left bronchus to left auricular pressure. My own experience has included examples of neither of these, with the exception of one doubtful case of radial asymmetry; but I have under observation at the present time a man with mitral stenosis and auricular fibrillation of some years' duration, whose left pupil is persistently smaller than the right, and by a good deal. It is possible in the screen to see that the auricle is enlarged in a backward direction. Moreover, he complains of a choking sensation at the root of the neck on exertion, a symptom by no means uncommon in mitral stenosis. In one case, that of a girl with mitral disease and great cardiac enlargement, actual dysphagia was complained of. Dr. Bergin and I examined this latter patient with the screen, and we found three facts of interest. First, the auricle was enlarged and projected backwards in such a way that it could scarcely fail to compress the œsophagus (Fig. 8). Second, at this point the stream of bismuth down the œsophagus was distinctly diverted towards the right. Third, it was delayed

FIG. 8.

Appearances seen with fluorescent screen.
(Patient in right anterior oblique position.)
After Holzknecht.

at the level of the cricoid cartilage. I do not attempt to explain the connection between these phenomena, but they do at least suggest that mitral stenosis may be a cause of dysphagia, and that this adds to the possibility of erroneous diagnosis of aneurysm in such cases.

Finally, the *conus arteriosus of the right ventricle* may become so hypertrophied in mitral stenosis that its pulsation may simulate that of an aneurysm. In a man of middle age whom I saw several times with mitral stenosis and total arrhythmia, there was a very definite area of strong ventricular-timed pulsation (Fig. 9) in the third left interspace—so strong and well-defined that it seemed too much to ascribe to the ordinary cause of pulsation at that spot, namely, hypertrophy of the conus arteriosus dexter—and the possibility of an aneurysm being present was several times considered. In one case of aneurysm springing from a sinus of Valsalva, the pulsation of the sac was easily visible in this very same area. However, we were unable to find any confirmatory evidence of aneurysm in the man with mitral stenosis by the ordinary methods or with the X-rays, and after his death in another town I heard from the pathologist

FIG. 9.

who made the *post-mortem* examination that there was no aneurysm.

To summarise. The clinical picture which is familiarly associated with aortic aneurysm may be imitated not only by that of mediastinal growth and inflammation, but also by simple dilatation of the aorta and by chronic rheumatic disease of the heart. In the former type of the disease the direct physical signs of aneurysm are imitated, in the latter an enlarged auricle may produce some of its pressure effects.

DISCUSSION.

The above paper was discussed at the meeting of the Bristol Medico-Chirurgical Society on January 14th.—Dr. MICHELL CLARKE stated that syphilitic aortitis itself was sufficient to cause aneurysm without the blood pressure being raised. He had seen many cases with normal blood pressure. But increased pressure was of importance in cases of dilated aorta as distinguished from aneurysm. It was remarkable how great the dilatation of the conus arteriosus might be in cases of mitral and tricuspid stenosis. The pulsations could be detected to the left of the sternum. A sign of dilatation of the left auricle was sometimes dulness in the left interscapular space.—Dr. Nixon said he had seen two autopsies on patients who had practical obliteration of the carotid and subclavian arteries at their origin from the aorta. One of the patients had a radial pulse which was difficult to explain.—The President alluded to the value of paralysis of the left recurrent laryngeal nerve as a symptom of aneurysm in the absence of other pathological conditions in the neck. Many cases of aneurysm had been correctly diagnosed largely on the basis of this sign. He looked upon paralysis of the recurrent laryngeal, together with œsophageal obstruction as strong evidence of œsophageal new growth.—Dr. Coombs could not confirm the value of percussion in the detection of a dilated left auricle from behind. It was very difficult to explain the various disturbances of sensation from aneurysm.

REFERENCES.

[1] *Presse Méd.* 1913, p. 607.

[2] *Am. J. Med. Sc.*, 1911, cxli. 625.

## Figures and Tables

**Fig. 1. f1:**
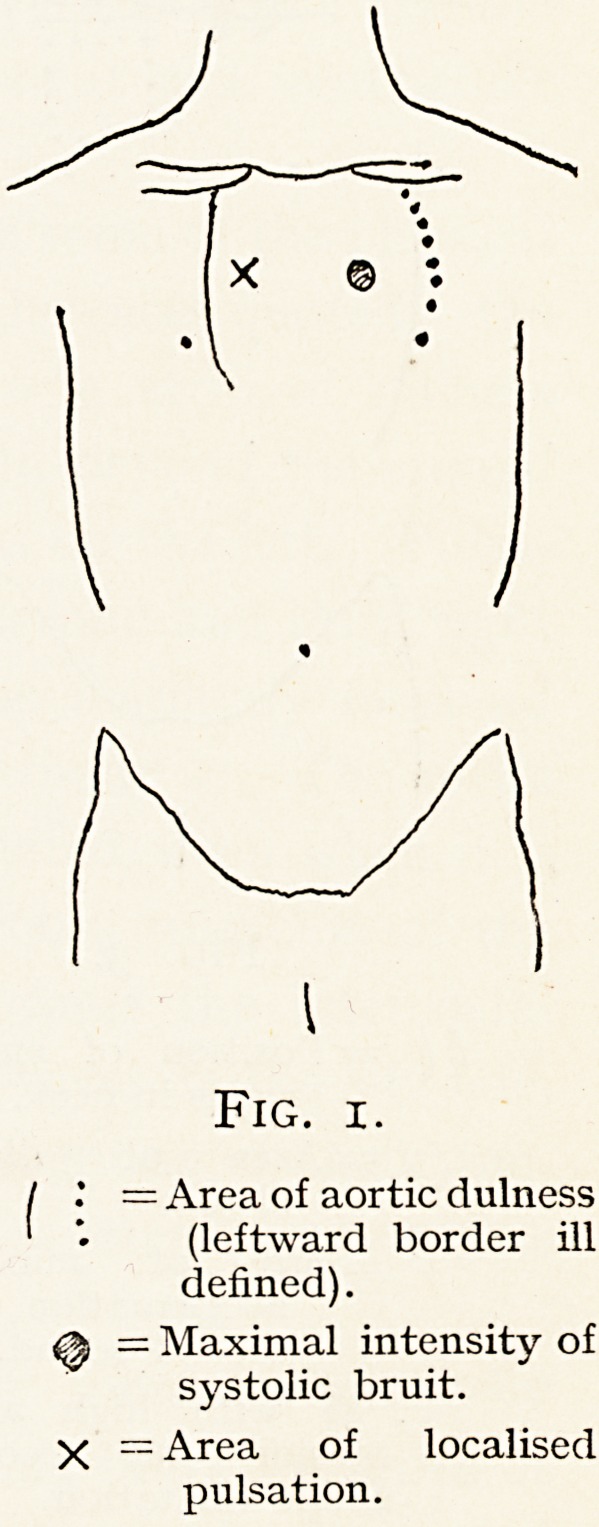


**Fig. 3. f2:**
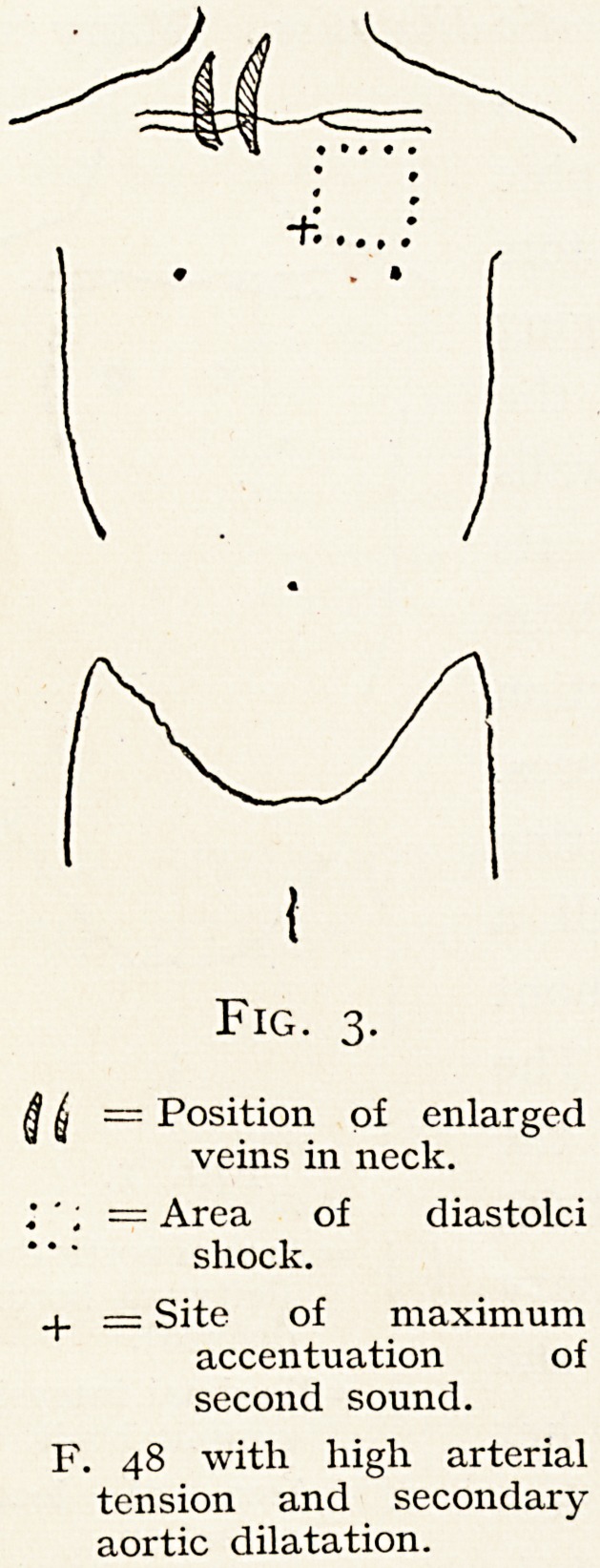


**Fig. 4. f3:**
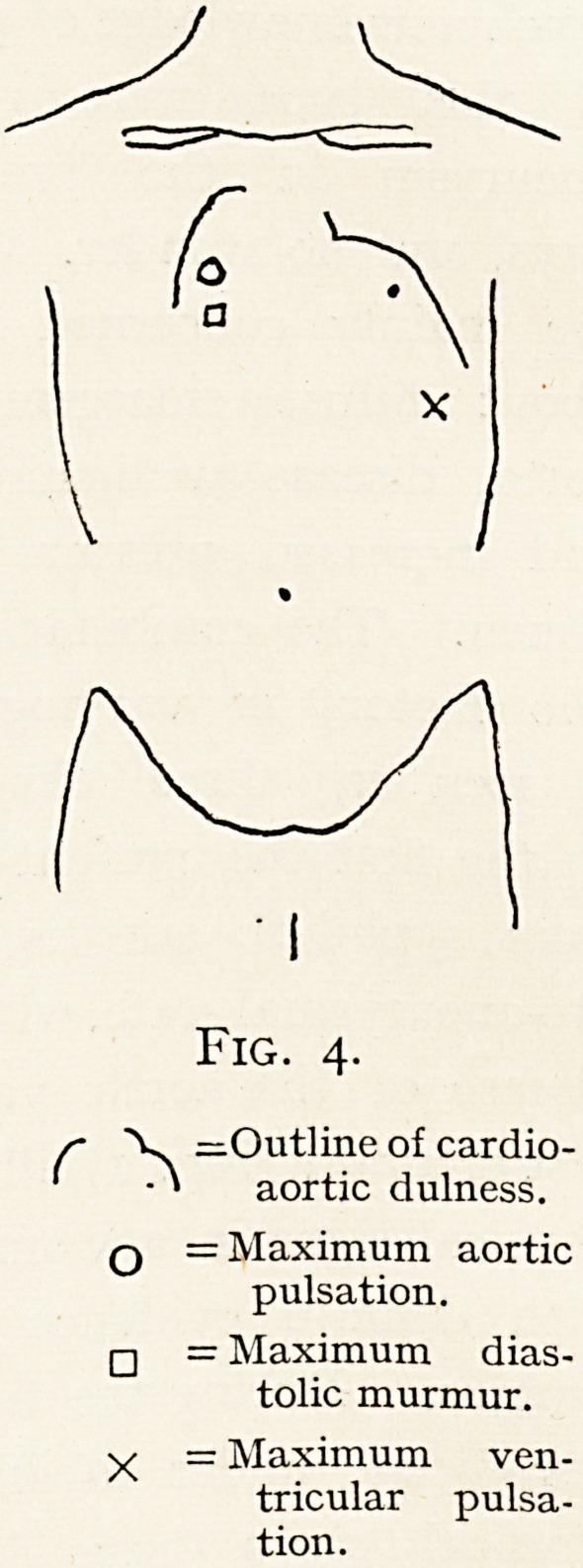


**Fig. 5. f4:**
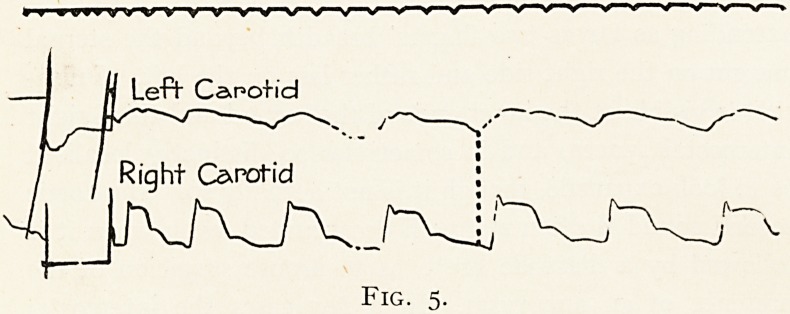


**Fig. 2. f5:**
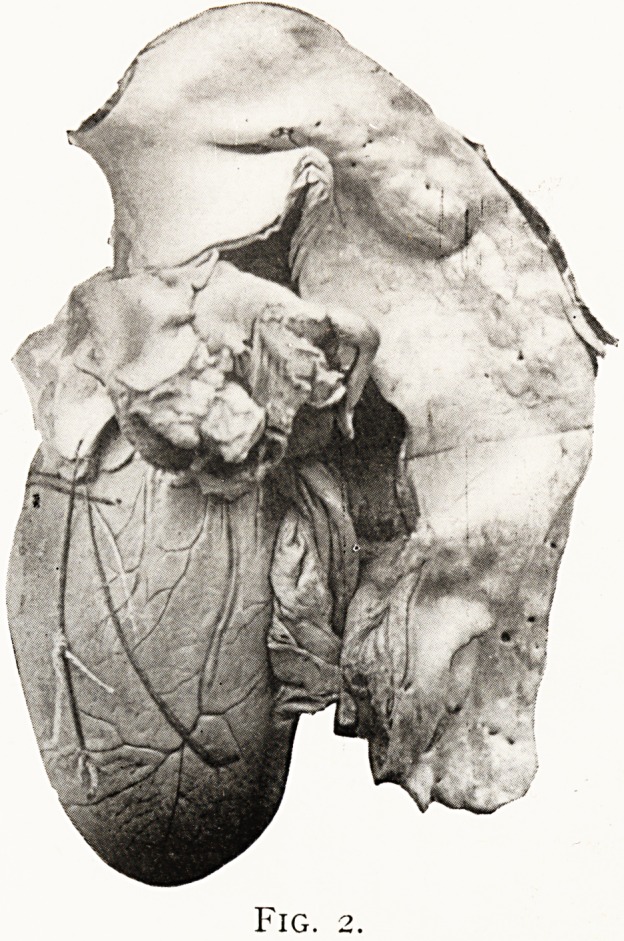


**Fig. 6. f6:**
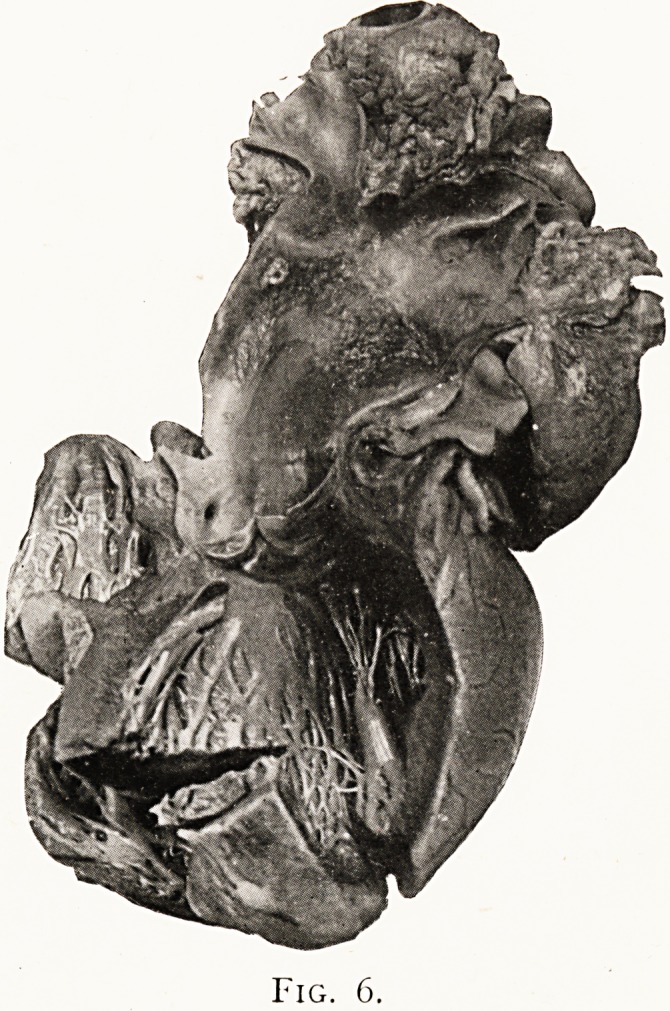


**Fig. 7. f7:**
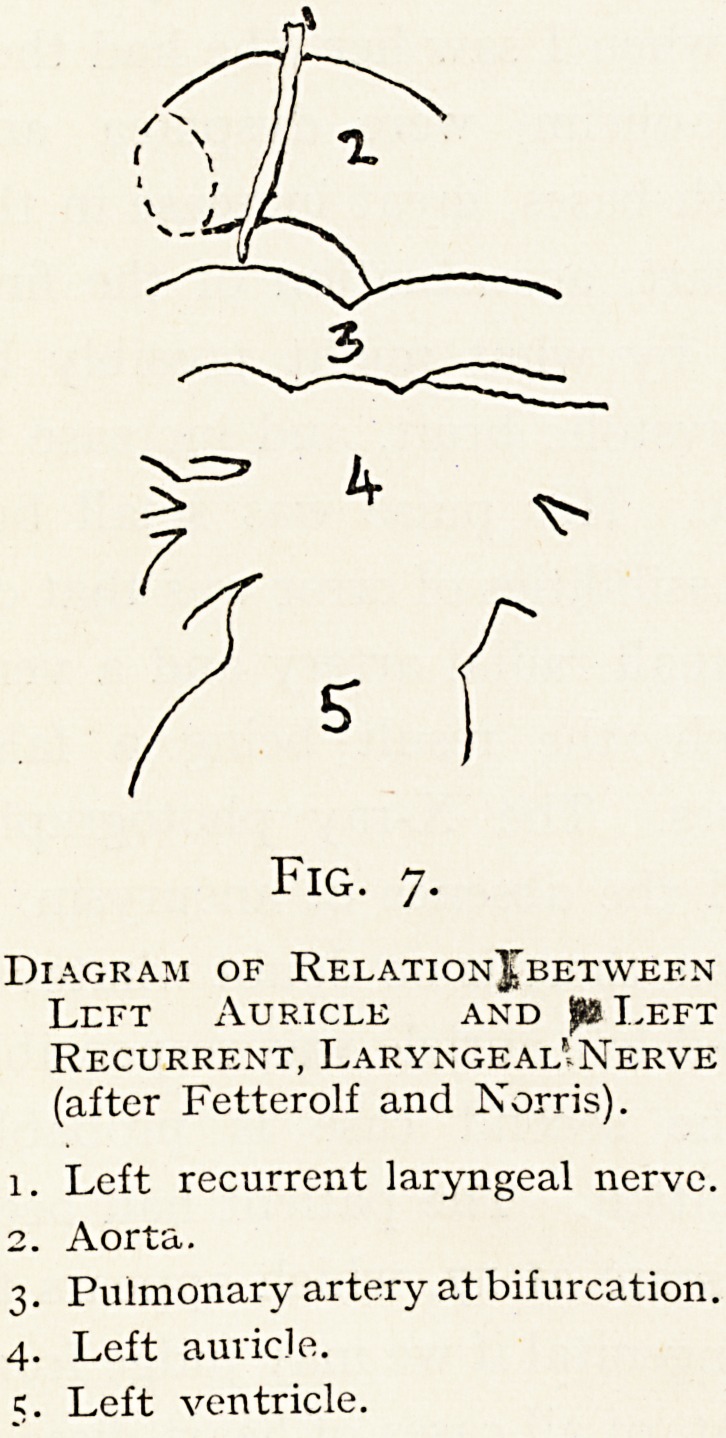


**Fig. 8. f8:**
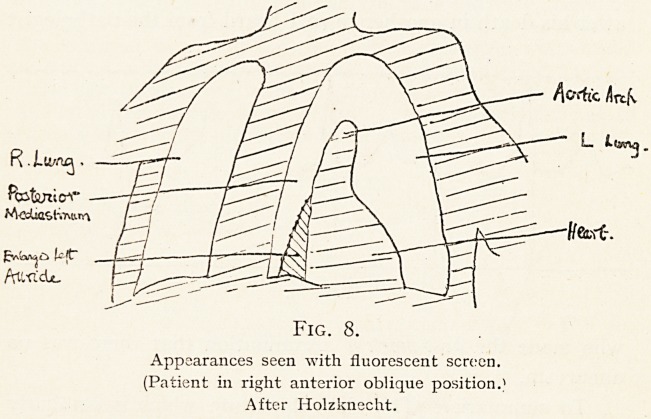


**Fig. 9. f9:**